# Antibacterial properties of silver and gold nanoparticles synthesized using *Cannabis sativa* waste extract against *Pseudomonas aeruginosa*

**DOI:** 10.1186/s42238-025-00272-0

**Published:** 2025-04-12

**Authors:** Jana Michailidu, Anna Miškovská, Irena Jarošová, Alena Čejková, Olga Maťátková

**Affiliations:** University of Chemistry and Technology, Technická 5, Praha 6, Prague, 166 28 Czechia

**Keywords:** Nanoparticles, Antimicrobials, Synthesis, Waste valorization, Cannabis

## Abstract

**Aims:**

The study aimed to explore the sustainable synthesis of metal nanoparticles using a green and eco-friendly resource. Specifically, it investigated the utilization of *Cannabis sativa* waste extract for the production of gold and silver nanoparticles, focusing on their antimicrobial activity against gram-negative bacteria, particularly *Pseudomonas aeruginosa* strains, which are significant in nosocomial infections.

**Methods:**

*Cannabis sativa* waste extract was employed to synthesize gold and silver nanoparticles through a green synthesis approach. The produced nanoparticles were characterized using transmission electron microscopy (TEM), atomic absorption spectrometry (AAS), X-ray diffraction (XRD), and Fourier transform infrared spectroscopy (FTIR). The antimicrobial efficacy of the synthesized nanoparticles was assessed through their minimum inhibitory concentration (MIC), minimum bactericidal concentration (MBC), and minimal biofilm inhibitory concentration (MBIC) against Pseudomonas aeruginosa, utilizing a microcultivation device, solid medium cultivation, and a metabolic activity assay in a polystyrene microtiter plate, respectively.

**Results:**

The TEM analysis revealed the size and morphology of the nanoparticles, while AAS confirmed their concentration. XRD provided insights into the crystalline structure, and FTIR analysis identified the molecular structure of the nanoparticle’s stabilizing layer. The synthesized nanoparticles showed significant antimicrobial activity against *Pseudomonas aeruginosa*, with determined MIC, MBC, and MBIC values of produced silver nanoparticles, showcasing their potential as effective antimicrobial agents.

**Conclusions:**

This study successfully demonstrated the synthesis of silver and gold nanoparticles using *Cannabis sativa* waste extract and highlighted their potent antimicrobial properties. It underscores the potential of utilizing plant waste extracts in sustainable nanomaterial synthesis and contributes to the fields of green nanotechnology and waste valorization within the circular economy. The findings also offer valuable insights into developing natural waste source-based antimicrobial agents.

## Introduction

In recent years, the exploration of eco-friendly and sustainable methods for nanoparticle production has gained significant attention within the scientific community (Adil et al. 2015; Jorge de Souza et al., 2019). Metal nanoparticles, with their unique physicochemical properties, have paved the way for advancements in various fields, including catalysis, electronics, medicine, and environmental science (Salem and Fouda 2021). However, conventional approaches of nanoparticle synthesis often involve utilization of hazardous chemicals and energy-intensive processes, leading to concerns regarding their environmental impact (Gan et al. 2012; Makarov et al. 2014; Zhang et al. 2008).

In this context, the search for alternative, green synthesis methods have become imperative. One promising avenue lies in the utilization of natural sources and by-products as reducing agents and stabilizers. *Cannabis sativa*, a versatile plant known for its medicinal and industrial applications, presents an intriguing opportunity for sustainable nanoparticle synthesis. Moreover, the use of waste from *Cannabis sativa* extraction for this purpose holds immense potential due to its availability, low cost, and minimal environmental impact.

Silver and gold nanoparticles have garnered considerable interest in the field of antimicrobial research due to their potent activity against a wide range of Gram-negative bacteria (El-Seedi et al. 2019; Hassan et al. 2018; Salleh et al. 2020). Silver nanoparticles, in particular exhibit strong antibacterial properties by interfering with various cellular components and processes. The small size and large surface area-to-volume ratio of silver nanoparticles allow for increased contact and interaction with bacterial cells, leading to disruption of the cell membrane, leakage of cellular contents, and induction of oxidative stress. These nanoparticles can also penetrate bacterial biofilms, which are often responsible for antibiotic resistance. Additionally, silver nanoparticles have been shown to inhibit key enzymes involved in bacterial metabolism, DNA replication, and protein synthesis, further compromising bacterial viability (Dakal et al. 2016; Gurunathan et al. 2014; Singh et al. 2018a).

Similarly, gold nanoparticles possess antimicrobial activity against Gram-negative bacteria through mechanisms such as membrane disruption and interference with bacterial adhesion and biofilm formation (Cui et al. 2012). The level of antibacterial efficacy of gold nanoparticles can be attributed to their size, shape, surface charge, and functionalization (Brown et al. 2012; Shamaila et al. 2016). Overall, the antimicrobial properties of silver and gold nanoparticles make them promising candidates for the development of novel therapeutic strategies against bacterial infections.

*Cannabis sativa* waste, obtained from various parts of the plant is typically discarded after the extraction of cannabinoids and other valuable compounds. However, recent studies have demonstrated that these waste materials contain a rich repertoire of bioactive compounds, including flavonoids, terpenes, and phenolic compounds, which possess inherent reducing and stabilizing properties (Bankar et al. 2010; Baruwati and Varma 2009; Castro et al. 2015; Dikshit et al. 2021; Iravani 2011). Harnessing these natural compounds can provide a green and efficient route for the synthesis of metal nanoparticles, while simultaneously valorising an otherwise unused resource.

A fundamental challenge when utilizing plant-derived waste in nanomaterial synthesis is the inherent variability in its chemical composition. The phytochemical profile of *Cannabis sativa* waste is influenced by several factors, including the genetic background of the plant, environmental conditions during cultivation (such as soil composition, temperature, and light exposure), and the extraction method employed (Namdar et al. 2018). These factors impact the relative abundance of secondary metabolites such as flavonoids, terpenes, and phenolic compounds, which are known to possess reducing and stabilizing properties in nanoparticle synthesis (Andre et al. 2016; ElSohly and Slade 2005). In particular, flavonoids and phenolic acids present in *Cannabis sativa* waste have been reported to act as natural reducing agents in green synthesis processes, contributing to the formation and stabilization of metallic nanoparticles (Beleggia et al. 2023; Iravani 2011). Given this variability, reproducibility in nanoparticle synthesis using *Cannabis sativa* waste may be influenced by batch-to-batch differences in extract composition, necessitating further investigation into standardization methods.

The objective of this article is to comprehensively explore the potential of *Cannabis sativa* waste extract as a sustainable and eco-friendly precursor for metal nanoparticle production. We aim to highlight the extraction of bioactive compounds from *Cannabis sativa* waste, and their subsequent utilization in nanoparticle synthesis. Furthermore, we will discuss the physicochemical properties of these metal nanoparticles synthesized using *Cannabis sativa* waste extract.

To evaluate the efficacy of these nanoparticles, their antimicrobial activity will be tested against different strains of *Pseudomonas aeruginosa*, a Gram-negative bacterium known for its resistance to antibiotics and its association with various infections. *Pseudomonas aeruginosa* is commonly found in hospital settings and poses a significant threat not only to immunocompromised patients but also to those with severe wounds, burns, or undergoing invasive procedures. Assessing the antimicrobial potential of the synthesized nanoparticles against *Pseudomonas aeruginosa* strains will provide valuable insights into their effectiveness as antimicrobial agents.

By leveraging the inherent properties of *Cannabis sativa* waste, we envision a sustainable and cost-effective approach towards nanoparticle synthesis, addressing both the environmental concerns associated with conventional methods and the growing demand for green technologies. This research holds significant promise for the development of novel nanomaterials with unique properties, opening doors for advancements in diverse fields and facilitating the transition to a more sustainable future.

## Materials and methods

### Materials for nanoparticle synthesis

The materials utilized for synthesis consisted of *Cannabis sativa* waste from butane mediated extraction. Additional materials included silver nitrate and tetrachlorauric acid obtained from Sigma Aldrich, ethanol (40%), and distilled water.

### Preparation of plant extract

To extract the desired components, a modified extraction method was employed according to (Rollová et al. 2020). The *Cannabis sativa* waste was homogenized using a blade blender, and 150 g of the homogenized canes were mixed with 600 mL of a 40% ethanol solution. This mixture underwent maceration for 24 h and was subsequently filtered through an 8 μm filter paper to remove impurities, followed by sterilization with a 0.2 μm filter. The resulting extract was stored at 7 °C for up to 7 days before further use in the synthesis process.

### Green synthesis of metal nanoparticles

The green synthesis method was employed for the production of silver and gold nanoparticles (AgNPs and AuNPs) using the ethanolic extract of *Cannabis sativa*, tetrachloroauric acid or a silver nitrate solution. Several reagent ratios were tested during the initial experiments. Firstly, experimental layout using a constant extract concentration of 10% and varying metal ion concentrations from 0.5 to 5.0 mM was employed, and a second set of experiments with a constant AgNO_3_ and HAuCl_4_ content (1 mM) and varying extract concentrations from 1.5 to 15.0% was carried out. The reaction mixture was allowed to react for 48 h, and the resulting suspension of AgNPs and AuNPs was characterized using UV-Vis spectrophotometry. Based on these pilot experiments, the final reagent ratios for the antimicrobial testing stock dispersions were determined as 10% v/v of *Cannabis sativa* extract with a 4.5 mM solution of silver nitrate (for AgNPs), and 10% v/v of *Cannabis sativa* extract with a 2 mM solution of tetrachloroauric acid (for AuNPs).

### Nanoparticle characterization

For the characterization of synthesized nanoparticles, analyses were performed including transmission electron microscopy (TEM), atomic absorption spectrometry (AAS), X-ray diffraction (XRD), and Fourier transform infrared spectroscopy (FTIR). In the TEM method, the nanoparticles were kept in dispersion form in the extract, while the AAS method utilized the supernatant obtained by centrifuging the nanoparticles in the extract. The nanoparticles were centrifuged, resuspended, and subsequently lyophilized for the XRD and XPS methods. TEM characterization was conducted using the EFTEM Jeol 2200 FS instrument, followed by image analysis using ImageJ software. AAS characterization was performed using the AGILENT 280 FS AA instrument, while XRD characterization utilized the PANalytical X’Pert PRO diffractometer with CuKα radiation. The elemental composition of the nanoparticle surface was investigated using XPS on the ESCA ProbeP instrument. The molecular structure of the stabilizing layer on the nanoparticles was analyzed using FTIR on the Nicolet 6700 instrument.

### Microbial strains and growth media

The study utilized various strains of *Pseudomonas aeruginosa*, including PAO1, ATCC 10,145, ATCC 15,442, DBM 3081, and DBM 3777. Glycerol cryopreserves of these strains were stored at -70 °C. Prior to each experiment, all *P. aeruginosa* strains were precultivated in a Luria-Bertani (LB) liquid medium at 37 °C for 24 h to achieve the exponential growth phase. The cultivation was carried out in Erlenmeyer flasks with a volume of 100 mL and agitation at 150 rpm.

The selection of *Pseudomonas aeruginosa* strains used in this study was based on their clinical relevance, availability in our laboratory, and their varying resistance profiles. The PAO1 strain is widely used as a model organism for studying *P. aeruginosa* pathogenicity and antimicrobial resistance (Stover et al. 2000). The ATCC 10,145 and ATCC 15,442 strains are reference strains frequently used in antimicrobial susceptibility testing and biofilm studies (Mah et al. 2003). The DBM 3081 strain is a soil isolate, while DBM 3777 is an industrial strain used for rhamnolipid production. These strains were included to represent a broader spectrum of *P. aeruginosa* phenotypes and assess the inhibitory effects of the synthesized nanoparticles across different ecological and functional variants of this species. By incorporating both clinical and environmental strains, as well as a biofilm-producing industrial strain, we aimed to evaluate the potential variability in antimicrobial activity and the broader applicability of our findings.

### Evaluation of minimal inhibitory concentration (MIC)

To assess the antimicrobial effects of the biosynthesized AgNPs and AuNPs against planktonic cells, a microcultivation device (Bioscreen C, Finland) and the microdilution method were employed (Sharma et al. 2010). The *P. aeruginosa* cells were cultivated in a microtiter plate for 24 h, with different concentrations of AgNPs and AuNPs added to each well. The experiment was conducted in 10 parallels and 3 independent repetitions. The wells contained LB medium (160 µL), inoculum (30 µL, OD_600_ = 0.1), and varying ratios of phosphate buffer saline solution and AgNPs (tested concentrations being 1.0, 2.1, 4.2, 6.3, 8.4, 10.5 mg/L) or AuNPs (tested concentrations being 1.2, 2.4, 4.8, 7.2, 9.5, 11.9 mg/L). Control parallels consisted of LB medium, inoculum, and phosphate buffer saline solution. The growth curves obtained from the microcultivation device data were used to determine the minimal inhibitory concentration (MIC_80_). MIC_80_ represents the lowest concentration of antimicrobial agent that resulted in 80% inhibition of growth compared to the control after overnight cultivation (Serra et al. 2018).

### Evaluation of minimal bactericidal concentration (MBC)

The bactericidal effect of biosynthesized nanoparticles was assessed by performing subsequent solid medium cultivations using LB agar plates. Planktonic cells cultivated according to the method mentioned in the MIC experiment method (see above) were transferred to the agar plates. The *P. aeruginosa* cells were cultivated for 24 h in a microtiter plate with different percentages (v/v) of AgNPs and AuNPs derived from their respective MICs (1x MIC, 2x MIC, 5x MIC). The experiment was conducted in 10 parallels and 3 independent repetitions. Control parallels contained LB medium, inoculum (30 µL, OD_600_ = 0.1), and phosphate buffer saline solution. A total of 10 µL of cell suspension representing each nanoparticle concentration and the control were inoculated onto an LB agar plate and cultivated for additional 24 h. The minimal bactericidal concentration (MBC) was determined as the concentration that resulted in the growth of fewer than five colonies (indicating > 99% killing) through the evaluation of growth on solid medium, following the method of Rahal and Simberkoff (Rahal and Simberkoff 1979).

### Evaluation of minimal biofilm inhibitory concentration (MBIC)

For the assessment of minimal biofilm inhibitory concentration (MBIC), cultivation was carried out in polystyrene 96-well microtiter plates. The *P. aeruginosa* cells were cultivated for 24 h in the presence of different percentages (v/v) of AgNPs and AuNPs derived from their respective MICs, ranging from 0% v/v to 3x MIC. Each well contained an inoculum (210 µL, OD_600_ = 0.8) with varying ratios of phosphate buffer saline solution and AgNPs and AuNPs (70 µL). Control parallels consisted of LB medium, inoculum (30 µL, OD_600_ = 0.8), and phosphate buffer saline solution. The experiment was performed in 8 parallels and 3 independent repetitions. The microtiter plate was covered with a lid and incubated at 37 °C and 150 rpm for 24 h.

### MTT assay

To measure the metabolic activity of the biofilm, a 3-(4,5-dimethylthiazol-2-yl)-2,5-diphenyltetrazolium bromide (MTT) reduction assay was conducted, following the method of Sabaeifard et al. (Sabaeifard et al. 2014). The wells were washed with saline and then treated with glucose solution (60 µL, 57.4 mg/mL) and MTT solution (50 µL, 1 mg/mL). The microtiter plate was covered and incubated in the dark at 37 °C and 150 rpm for 1 h. After incubation, wash solution (100 µL) was added to each well, and the plate was agitated for 30 min at room temperature. The wash solution comprised a mixture of dimethylformamide, phosphate buffer saline solution, acetic acid, and sodium dodecyl sulfate. Following agitation, the color intensity was measured at 570 nm using a microtiter plate spectrophotometric reader (Sunrise reader, Tecan, Switzerland). The experiment was performed in eight parallels and 3 independent repetitions. The minimal biofilm inhibitory concentration (MBIC_50_ and MBIC_90_) was determined as the lowest concentration of the nanoparticle suspension that inhibited biofilm formation by 50% and 90%, respectively, compared to the control (Shin and Eom 2019).

### Assessment of antimicrobial activity of *Cannabis sativa* extract

To ensure that the observed antimicrobial effects were attributable to the nanoparticles rather than the *Cannabis sativa* extract itself, a control experiment was performed using an extract-only condition. The *Cannabis sativa* extract was applied at a concentration equivalent to the highest extract content used in the nanoparticle suspensions, corresponding to the maximum tested nanoparticle concentration (10% v/v). The antimicrobial assessment was conducted under identical conditions as the nanoparticle experiments, including the use of the Bioscreen C system for planktonic growth evaluation and the microtiter plate assay for biofilm formation analysis.

The results demonstrated that the *Cannabis sativa* extract alone did not exhibit a significant inhibitory effect on *Pseudomonas aeruginosa* growth or biofilm formation, as bacterial optical density (OD_600_) and metabolic activity remained comparable to those of the untreated controls.

### Statistical analysis of nanoparticle effects on *Pseudomonas aeruginosa* strains

All statistical analyses were conducted in Python using the statsmodels and scipy libraries. The effects of silver (Ag) and gold (Au) nanoparticles on planktonic and adhering *Pseudomonas aeruginosa* strains were assessed using optical density (OD) measurements and MTT-based metabolic activity assays, respectively.

For the planktonic growth experiment, OD values were analyzed using a one-way ANOVA to determine whether nanoparticle concentration significantly affected bacterial growth. A separate two-way ANOVA was performed to assess whether there was a strain-specific response to different nanoparticle concentrations by including strain × concentration interaction effects. Tukey’s Honestly Significant Difference (HSD) post-hoc test was conducted to identify which concentration groups significantly differed from the control.

For the biofilm inhibition (MBIC) experiment, the relative metabolic activity of biofilm cells was calculated as a percentage of the control. This dataset was analyzed using one-way ANOVA, followed by Tukey’s HSD test to determine whether specific concentrations led to statistically significant metabolic inhibition.

Statistical significance was set at *p* < 0.05 to assess the effects in both assays.

## Results

### Synthesis and activity of silver nanoparticles produced using cannabis sativa waste extract

**Fig. 1:**
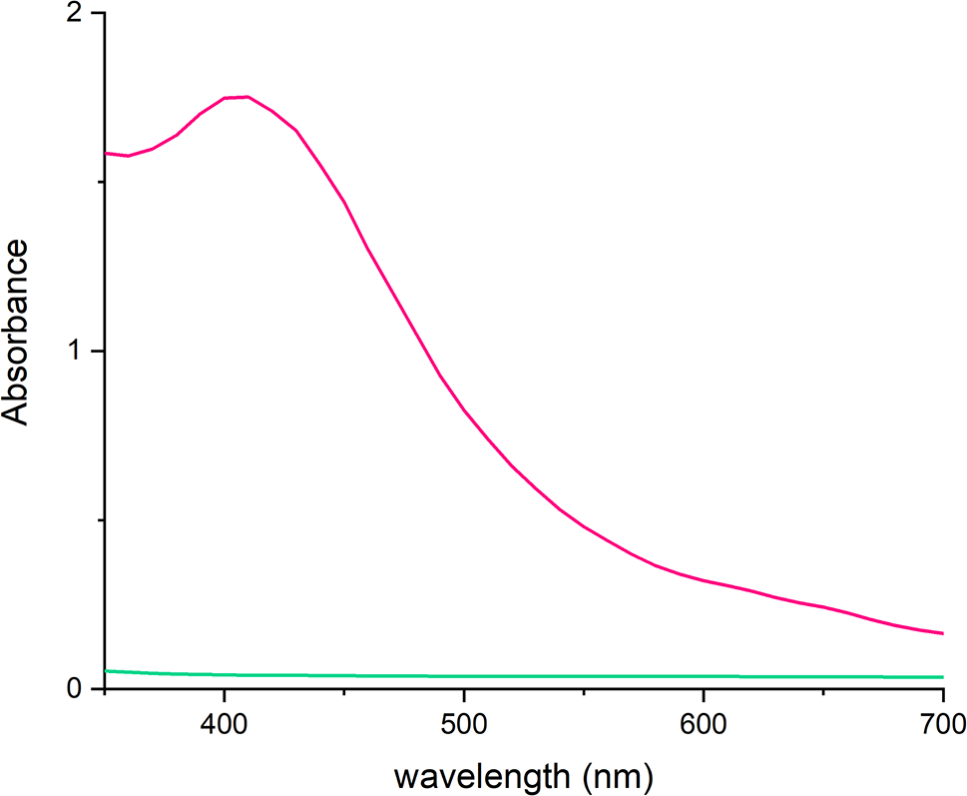
UV-Vis absorption spectra of AgNPs prepared using Cannabis sativa waste extract, pink – AgNPs, turquoise – plant extract. The data show a distinctive peak at wavelenghts characteristic for silver nanoparticles between 400 and 450 nm

Silver nanoparticles were successfully prepared using an extract from *Cannabis sativa* waste. Their formation was initially monitored by changes in visible light absorbance, and UV-Vis spectroscopy confirmed the successful synthesis. The presence of AgNPs was confirmed through UV-Vis spectrophotometry, showing peak absorbance at 450 nm due to plasmon resonance (see Fig. 1).

**Fig. 2:**
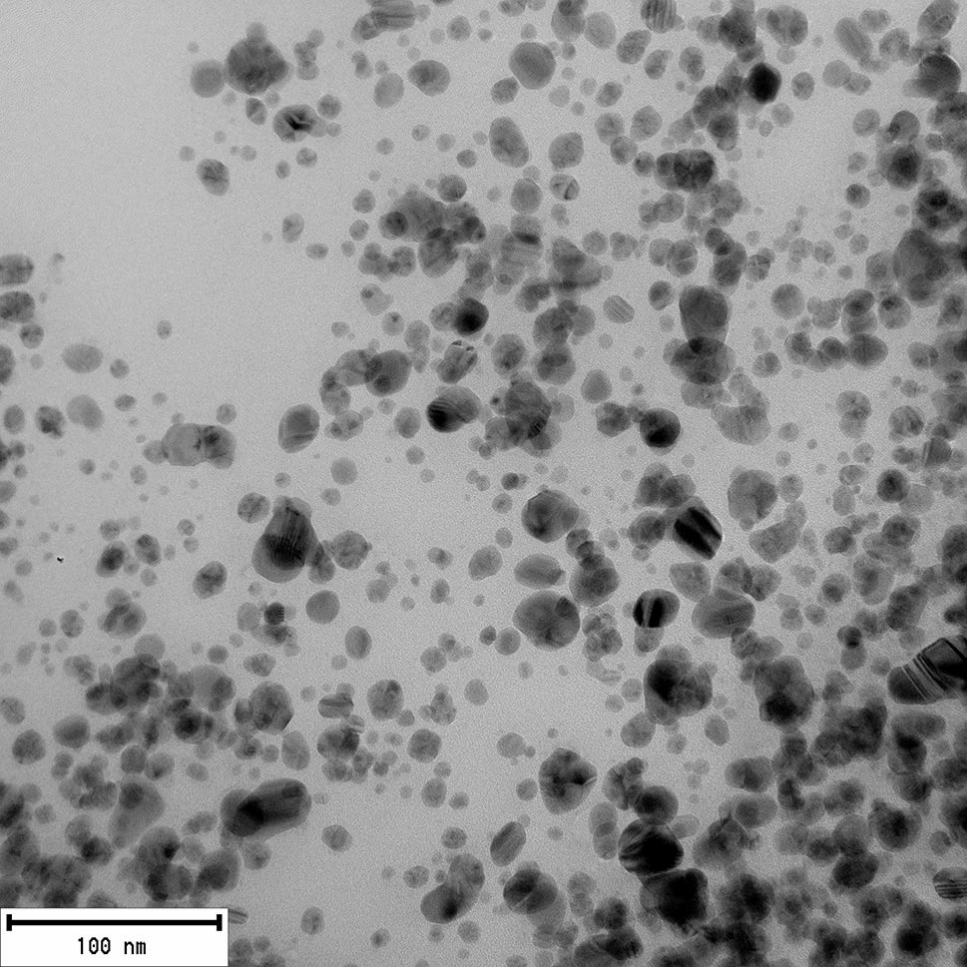
Transmission electron microscopy (TEM) photograph of AgNPs synthesized using Cannabis sativa extract, predominantly showing spherical shapes. The images provide a visual representation of nanoparticle morphologies, with the AgNPs exhibit predominantly spherical shapes, with occasional ellipsoid particles

**Fig. 3:**
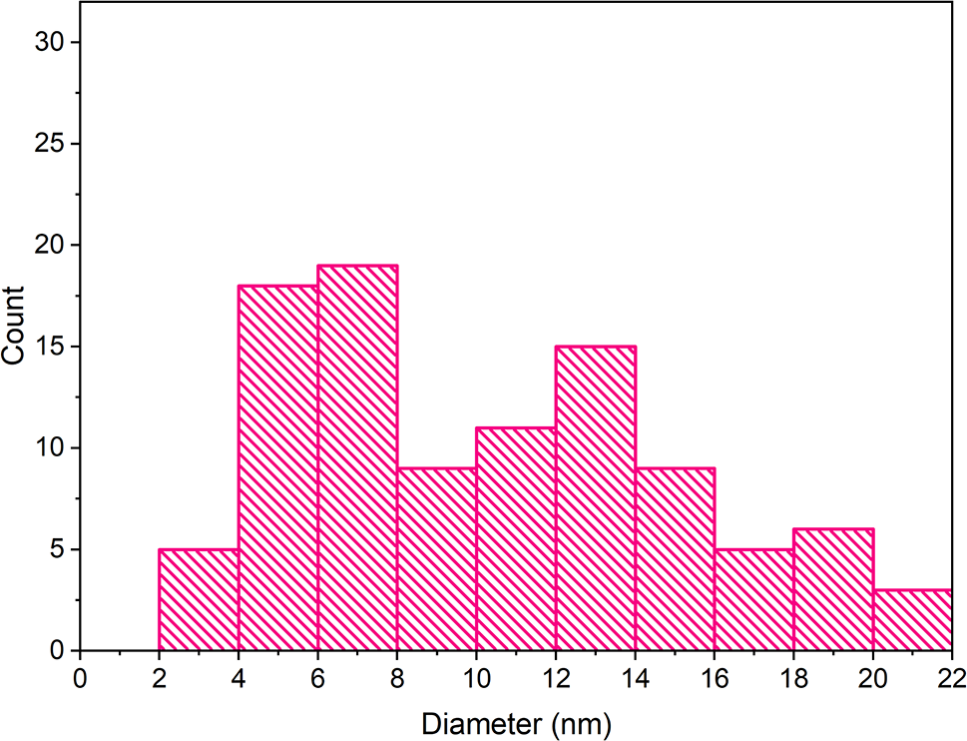
Histogram representing the size distribution of AgNPs synthesized using Cannabis sativa waste extract, in nanometers (nm), where most nanoparticles exhibited sizes between 3 and 21 nm

The concentration of the synthesized AgNP dispersion was determined as 334.4 mg/L using atomic absorption spectrometry (AAS). Transmission electron microscopy (TEM) revealed that the silver nanoparticles exhibited predominantly spherical or ellipsoidal morphology (see Figs. 2). Image analysis using the photographs resulted in a size distribution histogram of the produced nanoparticles. The average nanoparticle size ranged from 3 to 21 nanometers, with the highest frequency (27%) observed between 5 and 7 nm (see Fig 3).

**Fig. 4:**
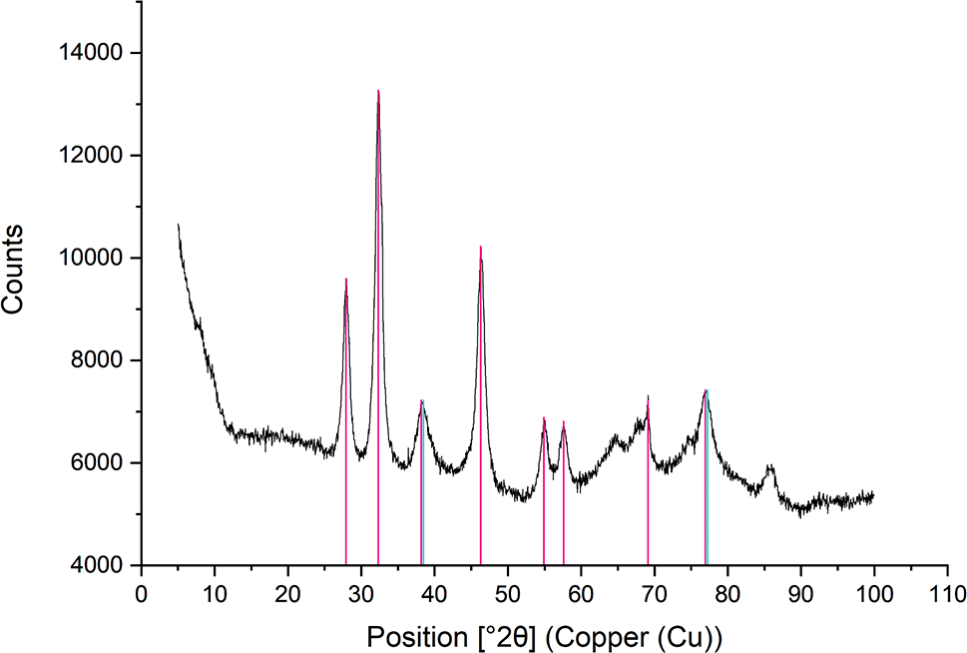
X-ray diffractogram of AgNPs synthesized using Cannabis sativa waste extract. The x-axis represents the diffraction angles (2θ), while the y-axis denotes the intensity of the diffracted X-rays. The diffractograms confirmed the presence of metallic silver and chlorargyrite in the tested sample

X-ray diffractograms of the AgNPs exhibited diffraction peaks at 2θ = 38.3°, corresponding to the (111) plane of metallic silver, and peaks at 2θ = 27.9°, 32.3°, 46.3°, 54.9°, 57.5°, and 76.8°, corresponding to the (111), (200), (220), (311), (222), and (420) planes of silver chloride (see Fig. 4). X-ray photoelectron spectroscopy confirmed the presence of carbon, oxygen, and silver on the surface layers of the AgNPs, with relative compositions of 31.0%, 39.6%, and 29.4%, respectively.

**Fig. 5:**
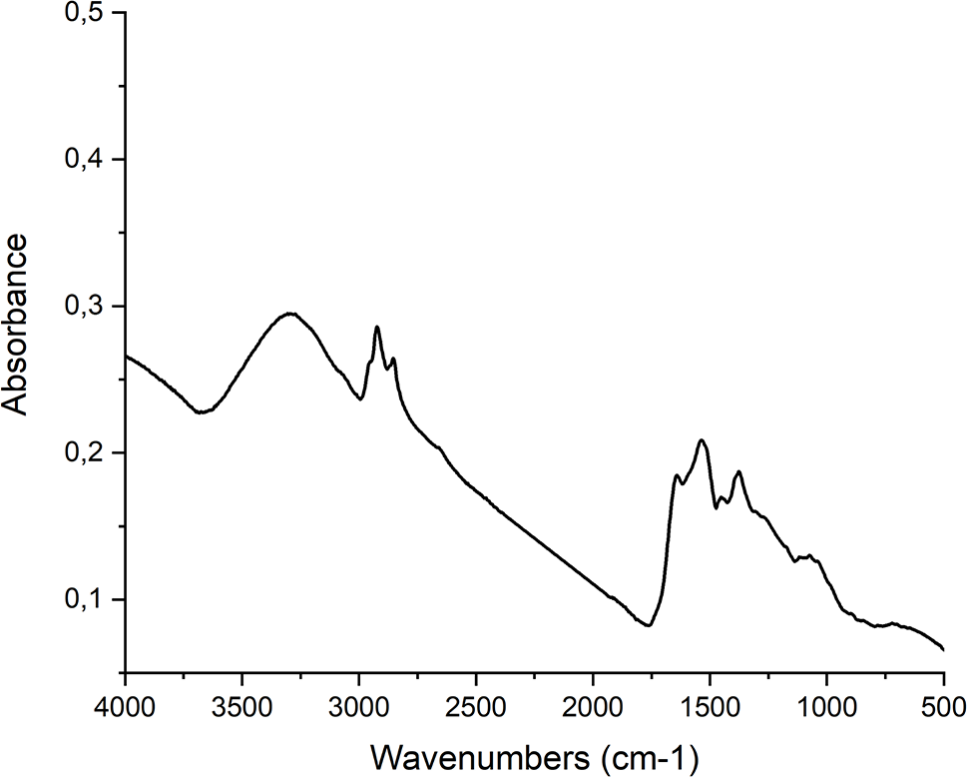
Fourier Transform Infrared (FTIR) Spectrum of Bimetallic Nanoparticles. This figure displays the FTIR spectrum of AgNPs synthesized using Cannabis sativa waste extract. The x-axis represents the wavenumber (in cm⁻¹), and the y-axis denotes the absorbance. Each peak in the spectrum corresponds to a specific vibrational mode of a functional group present in the nanoparticles. FTIR spectroscopy reveals functional groups on the surfaces of AgNPs synthesized using Cannabis sativa extract

Fourier-transform infrared spectroscopy analysed the functional groups in the stabilizing layer of AgNPs (see Fig. 5). Absorption bands at 3305 cm^-1^ indicated the presence of O–H bonds, while the combined presence of vibrational bands at 1641 cm^-1^ confirmed the presence of C = C bonds and increased absorption bands at 1536 cm^-1^ and 1050 cm^-1^ indicated the presence of C–O bonds. Absorption bands at 3305 cm^-1^ and 2853 cm^-1^ indicated the presence of primary and secondary amine vibrations, while bands at 3305 cm^-1^ and 2922 cm^-1^ confirmed the presence of–OH group vibrations. Vibrations at 1641 cm^-1^ indicated the presence of protein carbonyl groups, and a broader absorption band at 1050 cm^-1^ may indicate vibrations of C–N bonds in aliphatic amines. Increased absorption at 700 cm^-1^ suggested the presence of C–Cl bond vibrations.

The investigation of the effects of AgNPs on planktonic cells of *P. aeruginosa* revealed a minimum inhibitory concentration (MIC_80_) ranging from 0.6 to 2.1 mg/L for all the studied strains. The bactericidal activity of AgNPs was also evaluated, and the minimum bactericidal concentrations ranged widely from 0.6 to 10.5 mg/L. Lastly, the inhibitory activity of AgNPs was examined, and the minimum biofilm inhibitory concentrations (MBIC_80_) found to be in the range of 2.1 to 9.6 mg/L (see Table 1 and 2).

**Table 1:** Antimicrobial properties of silver nanoparticles produced using *Cannabis sativa* waste extract against *Pseudomonas aeruginosa* cells

	MIC_80_	MBC	MBIC_80_
	(mg/L)		
*P. aeruginosa* DBM 3081	0.6	1.3	9.6
*P. aeruginosa* DBM 3777	2.1	10.5	9.6
*P. aeruginosa* ATCC 10,145	2.1	2.1	2.1
*P. aeruginosa* ATCC 15,442	1.1	0.6	8.4
PAO1	1.1	10.5	4.2

**Table 2:** Minimal bactericidal concentration to minimal inhibitory concentration ratio of silver nanoparticles produced using *Cannabis sativa* waste extract

	MBC: MIC_80_ ratio (type of effect)
*P. aeruginosa* DBM 3081	2.2 (bactericidal)
*P. aeruginosa* DBM 3777	5.0 (bacteriostatic)
*P. aeruginosa* ATCC 10,145	1.0 (bactericidal)
*P. aeruginosa* ATCC 15,442	0.5 (bactericidal)
PAO1	9.5 (bacteriostatic)

### Synthesis and activity of gold nanoparticles produced using Cannabis sative waste extract

**Fig. 6:**
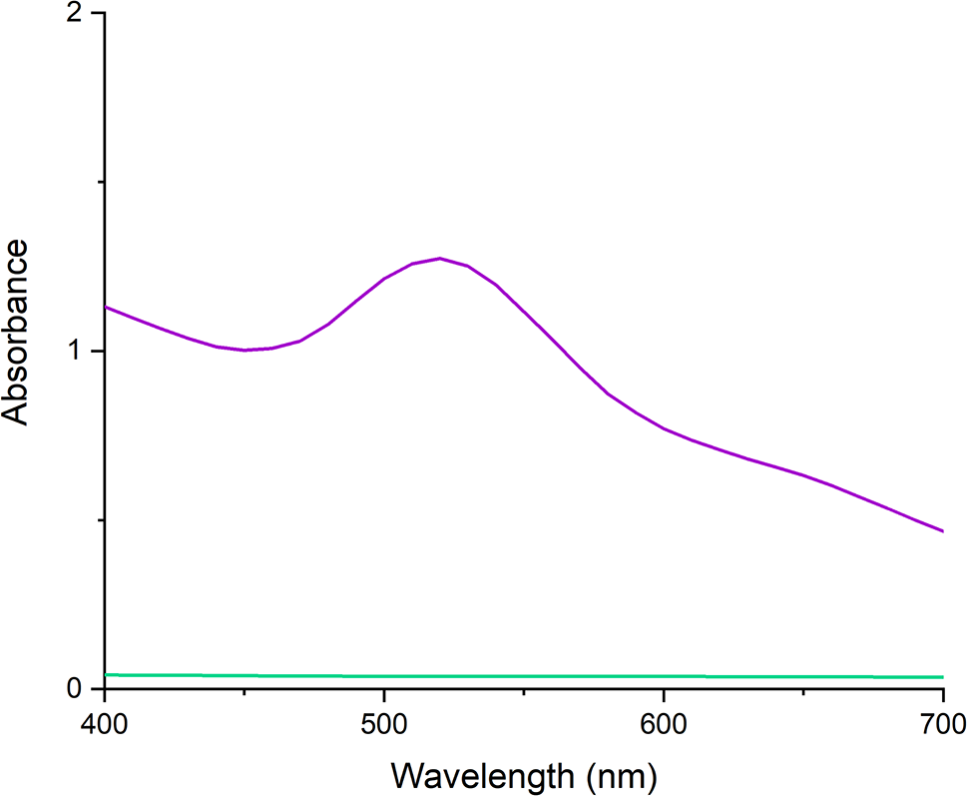
UV-Vis absorption spectra of Au NPs prepared using Cannabis sativa waste extract, purple – AuNPs, turquoise – plant extract. The data show a distinctive peak at wavelenghts characteristic for silver nanoparticles between 500 and 550 nm

Using an extract derived from *Cannabis sativa* waste, successful synthesis of gold nanoparticles was achieved. The formation process was initially tracked through changes in visible light absorption and later confirmed using UV-Vis spectroscopy. UV-Vis spectrophotometry confirmed the presence of AuNPs, with a prominent peak at 530 nm attributed to plasmon resonance (see Fig. 6).

**Fig. 7:**
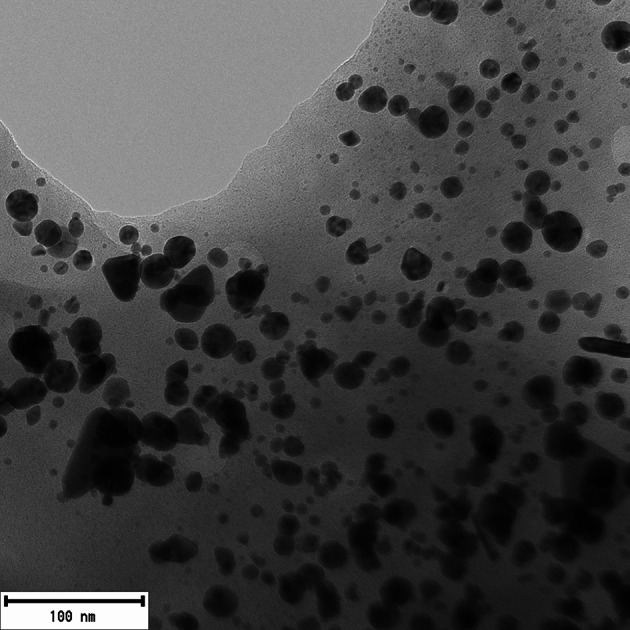
Transmission electron microscopy (TEM) photograph of AuNPs synthesized using Cannabis sativa extract, predominantly showing spherical shapes. The images provide a visual representation of nanoparticle morphologies, with the AuNPs exhibit predominantly spherical shapes, with occasional rod-like or triangular particles

**Fig. 8:**
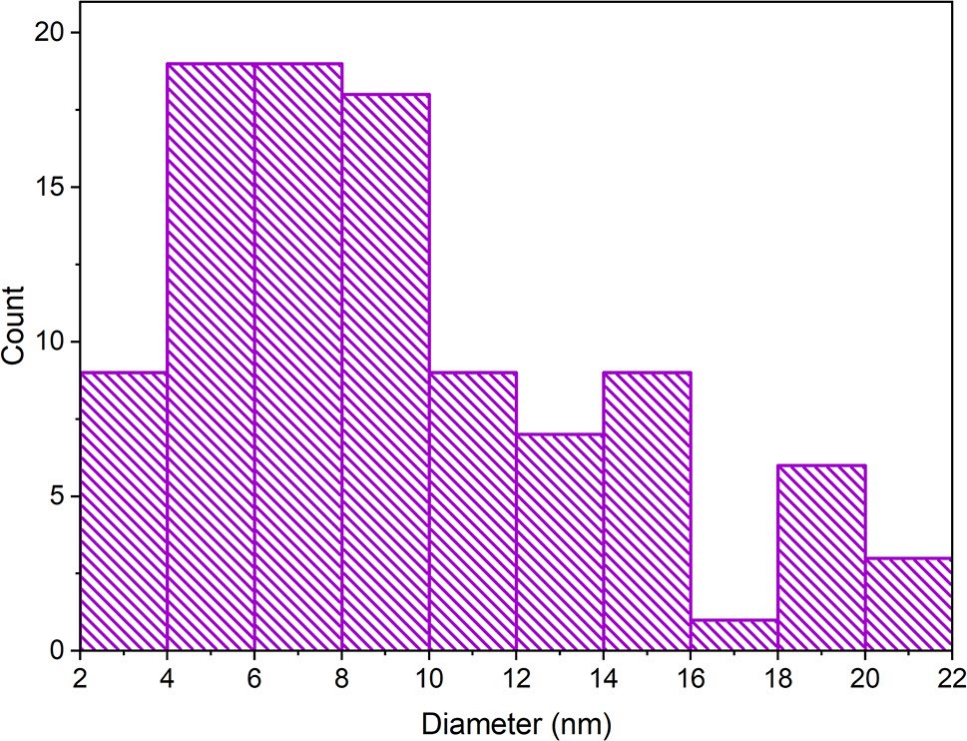
Histogram representing the size distribution of AuNPs synthesized using Cannabis sativa waste extract, in nanometers (nm), where most nanoparticles exhibited sizes between 2 and 21 nm

Atomic absorption spectrometry (AAS) determined the concentration of the synthesized AuNP dispersion as 381.9 mg/L. The morphology of AuNPs was examined using transmission electron microscopy (TEM), revealing predominantly spherical particles with occasional rod-like or triangular shapes (see Fig. 7). Image analysis of TEM photographs provided a size distribution histogram, indicating an average nanoparticle size ranging from 2 to 21 nanometers (see Fig. 8).

**Fig. 9:**
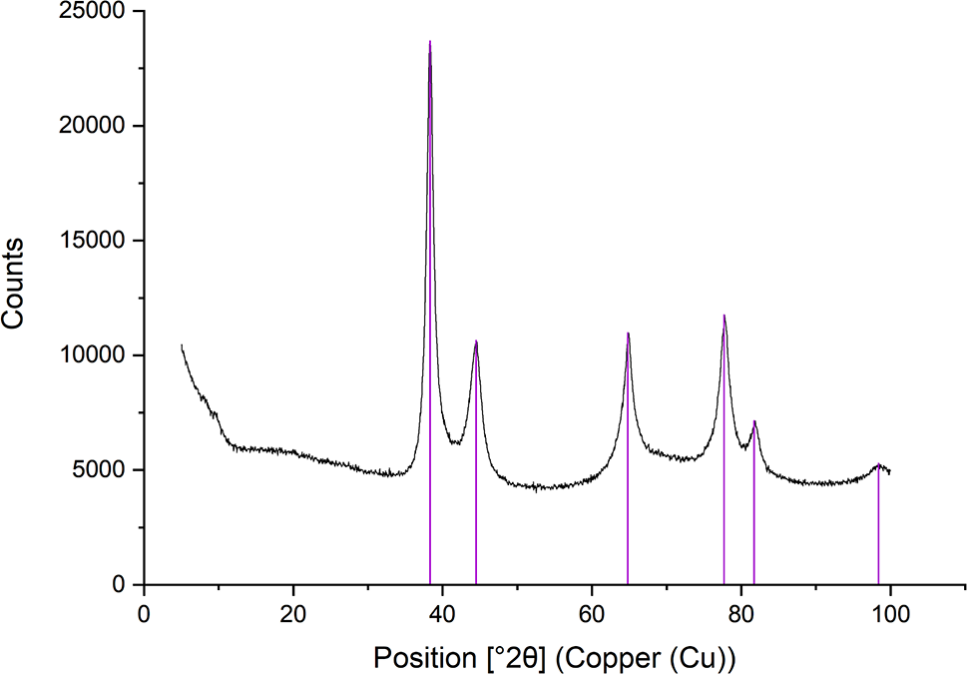
X-ray diffractogram of AuNPs synthesized using Cannabis sativa waste extract. The x-axis represents the diffraction angles (2θ), while the y-axis denotes the intensity of the diffracted X-rays. The diffractograms confirmed the presence of metallic gold in the tested sample

X-ray diffractograms exhibited characteristic diffraction peaks at 2θ = 38.3°, 44.5°, 64.8°, and 77.7°, corresponding to the (111), (200), (220), and (311) planes of metallic gold (see Fig. 9). X-ray photoelectron spectroscopy confirmed the presence of carbon, oxygen, and gold on the surface layers of the gold nanoparticles synthesized using *Cannabis sativa* extract, with relative compositions of 43.3%, 37.8%, and 18.9%, respectively.

**Fig. 10:**
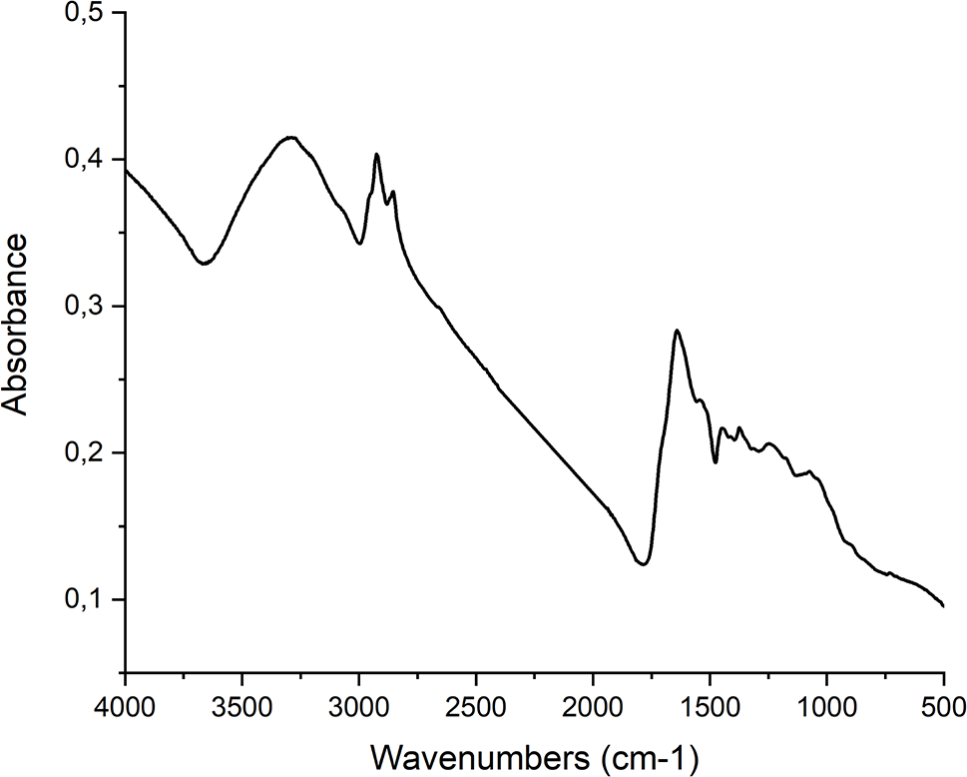
Fourier Transform Infrared (FTIR) Spectrum of Bimetallic Nanoparticles. This figure displays the FTIR spectrum of AuNPs synthesized using Cannabis sativa waste extract. The x-axis represents the wavenumber (in cm⁻¹), and the y-axis denotes the absorbance. Each peak in the spectrum corresponds to a specific vibrational mode of a functional group present in the nanoparticles. FTIR spectroscopy reveals functional groups on the surfaces of AuNPs synthesized using Cannabis sativa extract

Fourier-transform infrared spectroscopy (FTIR) analysis provided insights into the functional groups present in the stabilizing layer of the AuNPs (see Fig. 10). The broad absorption band observed at 3306 cm^-1^ confirmed the presence of O–H bonds. In combination with the absorption band at 1641 cm^-1^, the presence of C = C bonds was identified, while enhanced absorption at 1550 and 1050 cm^-1^ indicated the presence of C–O valence vibrations. The presence of C–H bonds was confirmed by the absorption bands observed at 2924 and 2855 cm^-1^, coupled with deformation vibrations at 1448 and 1374 cm^-1^. Additional information provided by FTIR analysis revealed that the absorption bands at 3306 and 2855 cm^-1^ confirmed the valence vibrations of primary and secondary amines. Moreover, the bands observed at 3306 and 2924 cm^-1^ corroborated the valence vibrations associated with hydroxyl group bonds. The valence vibration observed at 1641 cm^-1^ indicated the potential presence of carbonyl groups, potentially contributing to the stabilization through participating proteins. The absorption band at 1050 cm^-1^ suggested the presence of valence vibrations associated with C–N bonds in aliphatic amines.

During the investigation of the effects of AuNPs on *P. aeruginosa* strains, minimum inhibitory concentrations (MIC_50_) were found for all studied strains, ranging from 59.6 to 155.1 mg/L. In experiments evaluating the activity of gold nanoparticles synthesized using *Cannabis sativa* extract, minimum biofilm inhibitory concentrations (MBIC_50_) were achieved only for two studied strains, DBM 3777 (24.6 mg/L) and PAO1 (6.8 mg/L) (see Table 3).

**Table 3:** Antimicrobial properties of gold nanoparticles produced using *Cannabis sativa* waste extract against *Pseudomonas aeruginosa* cells

	MIC_50_	MBIC_50_
	(mg/l)	
*P. aeruginosa* DBM 3081	83.6	not found
*P. aeruginosa* DBM 3777	59.6	24.6
*P. aeruginosa* ATCC 10,145	155.1	not found
*P. aeruginosa* ATCC 15,442	83.6	6.8
PAO1	83.6	not found

*NOT FOUND – the minimal biofilm inhibiting concentration was not found in the concentration range used

Statistical analysis confirmed that AgNPs significantly reduced planktonic growth and biofilm metabolic activity, while AuNPs had no statistically significant effect. In the MIC assay, a one-way ANOVA showed that AgNPs significantly inhibited bacterial growth (*p* < 0.001), with Tukey’s post-hoc test confirming that MICs caused significant reductions compared to the control (*p* < 0.001). A strain × concentration interaction effect was significant (*p* < 0.001), confirming that nanoparticle efficacy varied by strain. AuNPs did not significantly inhibit planktonic growth at any concentration (*p* > 0.05).

In the biofilm inhibition (MBIC) assay, AgNPs at MIC showed a strong inhibitory effect (*p* < 0.001). Tukey’s post-hoc test confirmed significant differences (*p* = 0.0001). AuNPs did not significantly inhibit biofilm metabolic activity at any concentration (*p* > 0.05). These results highlight that the produced AgNPs exhibit potent antimicrobial effects both planktonic and biofilm conditions, whereas AuNPs lack significant activity under these experimental conditions.

## Discussion

During literature analysis, it was found that experimental studies dealing with the synthesis of nanoparticles using plant extracts exhibit some problematic aspects resulting in low comparability between the studies and limited reproducibility. A significant portion of these studies lacks sufficient information about the synthesis itself, such as specific concentrations of individual reagents, description of reaction conditions, and others. Another common deficiency in most studies in this field is the absence of information regarding sample preparation for nanoparticle characterization using analytical methods. Moreover, the results of these analyses are, in some cases, insufficiently commented on and discussed. Another issue is the determination of the concentration of the resulting nanoparticle dispersion, which is not addressed at all in some studies (Maťátková et al. 2022).

The primary method for detecting the successful production of metal nanoparticles, alongside visible colour changes in the dispersion within the visible spectrum, is undoubtedly UV-Vis spectrophotometry. With this technique, nanoparticles are characterized by their absorbance peak caused by surface plasmon resonance. Silver nanoparticles produced using *Cannabis sativa* leaf extract, showed an absorbance peak at a wavelength of 450 nm (Singh et al. 2018b). A study investigating the influence of selected *Cannabis sativa* cultivars on silver nanoparticle production found that all used extracts produced nanoparticles with an absorption maximum at wavelengths ranging from 432 to 439 nm (Csakvari et al. 2021). In another study exploring the use of *Cannabis sativa* leaf extract for silver nanoparticle synthesis, the peak absorbance was found to be in the range of 420–450 nm, depending on the ratio of the reaction components (Chouhan and Guleria 2020). In this work, silver nanoparticles synthesized using extracts from *Cannabis sativa* also exhibited a peak absorbance at 450 nm. Thus, it can be summarized that, according to the literature, silver nanoparticles show absorbance peaks in the range of 420–454 nm, and our findings fall within higher values within this range. A higher wavelength in surface plasmon resonance can signify not only larger nanoparticle diameters but also potentially greater aggregation or changes in the overall nanoparticle morphology. This could suggest that the *Cannabis sativa* extract used in our synthesis may induce specific structural properties or interactions leading to these larger or aggregated particles.

Gold nanoparticle production using *Cannabis sativa* extract was investigated in the study by Singh et al. (2018a, b, c), and its success was confirmed, among other methods, by UV-Vis spectrophotometry. The peak absorbance was observed at a wavelength of 550 nm (Singh et al. 2018b). In another study utilizing *Cannabis sativa* extract, absorbance peaks of nanoparticles formed by mixing different ratios of reaction components were compared. These values ranged from wavelengths of 548 to 551 nm (Hameed et al. 2020). A lower peak absorbance value of 538 nm was measured in a separate study using *Cannabis sativa* leaf extract (Chang et al. 2021). The spectra of gold nanoparticles produced in this study using plant extracts from *Cannabis sativa* exhibited absorption maximum at a wavelength of 530 nm. Our findings for gold nanoparticles fell within the lower end of the published range of absorbance peaks (530–551 nm). Lower wavelengths in surface plasmon resonance generally correlate with smaller nanoparticle diameters or more spherical shapes. This could be a result of the specific reductive properties of the *Cannabis sativa* extract used or the conditions under which synthesis was conducted, underscoring the intricate relationship between the biological reducing agents and nanoparticle characteristics.

Transmission electron microscopy can provide valuable information about the morphology of metal nanoparticles, and when combined with image analysis, it offers an overview of individual nanoparticle sizes and their size distribution in the dispersion. According to available literature, the morphology of silver nanoparticles synthesized using *Cannabis sativa* extracts mainly showed a spherical shape, with sizes ranging from 20 to 40 nm and 13 to 25 nm. In a study with statistical size analysis, the average size of nanoparticles was identified as 26.5 nm, with a median size of 29.9 nm (Chouhan and Guleria 2020; Singh et al. 2018a, b, c). The conclusions of these studies align with the results of this work, where silver nanoparticles of spherical or ellipsoidal shape were identified during synthesis using *Cannabis sativa* extract, with diameters ranging from 3 to 21 nm. The smaller size of nanoparticles formed in this study compared to literature suggests that the shift towards higher wavelengths in our UV-Vis spectra may be more indicative of nanoparticle aggregation rather than an increase in individual particle size.

The morphology of gold nanoparticles produced using plant extracts is consistently described in the literature as a mixture of triangular, hexagonal, rod-like, and spherical shapes, which aligns with the morphology of nanoparticles produced in this work. Their sizes ranged from 5 to 40 nm. Gold nanoparticles produced in this study using *Cannabis sativa* extract exhibited a similar size distribution (2–21 nm) to gold nanoparticles created with *Dracocephalum kotschyi* leaf extract, which showed diameters ranging from 5 to 21 nm (Barai et al. 2018; Chahardoli et al. 2019; Chang et al. 2021; Singh et al. 2018a, b, c). In accordance with literature, our gold nanoparticles, synthesized using *Cannabis sativa* extract, displayed mixed morphology with triangular, hexagonal, rod-like, and spherical shapes, and their size distribution (2–21 nm) closely mirrored that of nanoparticles derived from *Dracocephalum kotschyi* leaf extract.

The specific description of methods for determining the concentration of metal nanoparticles in the produced dispersion is relatively scarce in the literature. Many experimental studies do not address the determination of nanoparticle concentration at all, and for subsequent experiments (e.g., antimicrobial activity tests), the input concentration of the metal precursor is used. This assumes 100% conversion of metal ions without knowledge of the synthesis process mechanism or actual nanoparticle yield (Maťátková et al. 2022). However, some studies do focus on determining the actual nanoparticle concentration in the dispersion.

In a study investigating the production of gold and silver nanoparticles using *Cannabis sativa* extract, the concentration was determined by inductively coupled plasma mass spectrometry (ICP-MS). This method analyzed individual particles and found nanoparticle counts ranging from 26 to 83 billion particles per milliliter, corresponding to concentrations between 0.7 and 4.5 mg/mL (Singh et al. 2018b). Another study on the synthesis of copper nanoparticles using *Magnolia kobus* leaf extract revealed a linear relationship between nanoparticle concentration measured by ICP-MS and the absorbance peak size caused by surface plasmon resonance (Lee et al. 2011). In this work, atomic absorption spectrometry was used to determine the concentration of the produced nanoparticles. After separating the nanoparticles from the dispersion using centrifugation, the concentration of metal ions in the resulting supernatant was measured, and the concentration of metal in the nanoparticles was determined by mass balance using the initial molar concentration of the metal precursor. Using this method, the concentration of silver nanoparticles was found to be 334.4 mg/L, and the concentration of gold nanoparticles was found to be 381.9 mg/L.

The elemental composition and internal structure of metal nanoparticles are commonly characterized using X-ray diffraction (XRD) and X-ray photoelectron spectroscopy (XPS) in the literature. In a study investigating the synthesis of silver nanoparticles using *Cyperus pangorei* extract, XPS confirmed the presence of carbon, nitrogen, oxygen, and silver, identified by peaks at 285.1, 399.0, 531.0, and 366.6 eV, respectively (Parvathiraja et al. 2021). Other studies examining synthesis using extracts from *Sophora japonica* or *Clerodendrum spendens* confirmed these results, detecting oxygen (531.6 eV), silver (368.2 and 369.4 eV), and carbon (284.7 eV) (Cheng et al. 2022; Jayakumar and Vedhaiyan 2019). Similar results were obtained in this work. In the dispersion of silver nanoparticles produced using *Cannabis sativa* extract, carbon (284.9 and 284.6 eV), oxygen (532.9 and 531.4 eV), and silver (368.5 and 367.8 eV) were detected, confirming the presence of both the metal and stabilizing organic molecules. In line with established literature, our findings similarly highlight the presence of key elements like carbon, oxygen, and silver, detected at closely aligned eV values. This consistency not only underscores the efficacy of our nanoparticle synthesis using *Cannabis sativa* extracts but also attests to the uniform integration of metal with stabilizing organic molecules, mirroring the trends observed in other plant-mediated syntheses.

Information about the internal structure of nanoparticles is further explored using XRD in the literature. Studies describing the synthesis of silver nanoparticles using extracts from *Santalum album* fruits, *Leptadenia reticulata* leaves, *Aloe vera*, or *Carya illinoinensis* revealed four peaks at 2θ angles of 38.3, 44.6, and 64.8°, corresponding to (1 1 1), (2 0 0), and (2 2 0) planes attributed to the presence of face-centered cubic metallic silver lattice (Javan bakht Dalir et al. 2020; Kumara Swamy et al. 2015; Mehta et al. 2017; Zhang et al. 2013). However, in our study using *Cannabis sativa* extract, the XRD spectrum revealed additional peaks at 2θ angles of 27.9, 32.3, 46.3, 54.9, and 76.8°, which not only confirm the presence of metallic silver but also suggest the formation of silver chloride, as indicated by the (1 1 1), (2 0 0), (2 2 0), (3 1 1), and (4 2 0) planes, a finding consistent with the study involving *Oedera genistifolia* (Okaiyeto et al. 2019).

Regarding gold nanoparticles, a study using *Phragmites australis* leaf extract identified the presence of gold through peaks at 84.1 and 87.8 eV in the XPS spectrum (El-Borady et al. 2021). In other studies, where gold nanoparticles were synthesized using *Persicaria salicifolia* leaf extract and *Selenicereus* costaricensis fruit extract, the presence of gold, oxygen, nitrogen, and carbon was confirmed through peaks at 84.1 and 87.8; 531.3 and 532.6; 399.9 and 284.6 and 286.3 eV, respectively, in the XPS spectra (Divakaran et al. 2019; Hosny and Fawzy 2021). Consistent with the literature mentioned above, this work also identified the presence of gold, oxygen, and carbon, with peaks at 84.0; 284.4; and 532.4 eV for *Vitis vinifera* extract and 84.3; 285.1; and 532.4 eV for *Cannabis sativa* extract. Aligned with existing literature, our findings confirmed the presence of gold, oxygen, and carbon at closely associated eV values. The almost parallel eV peaks identified in our work, further corroborate the recurring elemental patterns seen in plant-mediated gold nanoparticle syntheses, underscoring the reliability and consistency of green synthesis methods.

XRD analyses in studies using *Coleus aromaticus*,* Phragmites australis*,* and Jasminum auriculatum* extracts for gold nanoparticle production confirmed the presence of face-centered cubic metallic gold lattice. This was achieved by identifying peaks at 2θ angles of 38.3, 44.4, 64.7, and 77.5° corresponding to (1 1 1), (2 0 0), (2 2 0), and (3 1 1) planes (Balasubramanian et al. 2020; El-Borady et al. 2021). The results of this work confirm the findings described in the literature above, with identified peaks at 2θ angles of 38.3, 44.5, 64.8, and 77.7° corresponding to (1 1 1), (2 0 0), (2 2 0), and (3 1 1) planes of metallic gold. Our study aligns seamlessly with established literature, exhibiting nearly identical 2θ angle peaks characteristic of the face-centered cubic metallic gold lattice. The consistency between our detected peaks and those reported in prior research underscores the reproducibility and reliability of the methods employed for gold nanoparticle synthesis across diverse botanical extracts.

Using Fourier-transform infrared spectroscopy (FTIR), functional groups of biomolecules stabilizing the formed nanoparticles were further analysed. Studies examining dispersions of silver or gold nanoparticles synthesized using *Cannabis sativa* extract detected absorption bands at 3332 cm^-1^ indicating the presence of O-H groups, valence vibrations at 1741–1604 cm^-1^ indicating C = C bonds, and an increased absorption band at 1019 cm^-1^ indicating valence vibration of C-O bonds. Additionally, clear absorption bands of carbonyl group valence vibrations, potentially belonging to proteins, were observed at 1635 cm^-1^. Valence vibrations of C-N groups in amines were also observed at 1045 cm^-1^. In this work, the presence of O-H groups was confirmed, showing an absorption band at 3305 cm^-1^, as well as the presence of C = C bonds indicated by a valence vibration band at 1641 cm^-1^. Other identified groups included C-O bonds with absorption bands at 1536 and 1050 cm^-1^ and a broader absorption band of aliphatic amines at 1050 cm^-1^. For silver nanoparticles and bimetallic nanoparticles synthesized using *Cannabis sativa* extract in this work, the presence of valence vibration of C-Cl bonds with absorption at 700 cm^-1^ was detected. Identical results were achieved in a study examining the synthesis of silver nanoparticles using *Cannabis sativa*, where these vibrations were attributed to peaks at 769, 687, and 526 cm^-1^. The reducing and stabilizing properties were attributed to reducing sugars or proteins, while the purely reducing activity was likely due to terpenoids and flavonoids (Singh et al. 2018a, b, c). Our FTIR analyses, utilized to identify the functional groups of biomolecules stabilizing nanoparticles, agrees with prior research, notably captured the O-H groups, C = C bonds, C-O bonds, and aliphatic amines at comparable wavenumbers. Moreover, the distinctive presence of C-Cl bonds in our synthesized silver nanoparticles mirrors findings in other studies using *Cannabis sativa* extract, underscoring the consistent role of certain biomolecules such as reducing sugars, proteins, terpenoids, and flavonoids in nanoparticle synthesis and stabilization as outlined in literature mentioned above.

Studies investigating the inhibitory activity of metal nanoparticles synthesized using “green” methods exhibit significant variability, including properties of the produced nanoparticles and tested microorganisms. Similarly, regarding the characterization of biosynthesized nanoparticles, a considerable portion of the studies lacks crucial information, such as the specific microbial strain used, the nanoparticle concentration used for testing, or the exact experimental setup. In terms of experimental design, most studies utilize methods like disk diffusion or well diffusion (Maťátková et al. 2022). Consequently, there is considerable variation in the results presented in these studies, leading to differences in the activity of the specific nanoparticles used.

The inhibitory activity of silver nanoparticles against *Pseudomonas aeruginosa* has been studied in various research works. In one study using silver nanoparticles synthesized from *Rheum palmatum* root extract, the minimum inhibitory concentration (MIC) against *Pseudomonas aeruginosa* ATCC 27,853 was determined as 15 mg/L. Silver nanoparticles produced using *Phyllanthus amarus* extract exhibited MIC values ranging from 6.25 to 12.5 mg/L against clinical isolates of *P. aeruginosa*. Another study utilizing silver nanoparticles synthesized with *Holarrhena pubescens* bark extract found MIC values of 20–25 mg/L. In a study using silver nanoparticles and silver chloride synthesized from *Malva sylvestris* leaf extract, confirmed by XRD analysis, MIC values for two *P. aeruginosa* strains were found to be 15.6 and 62.5 mg/L. In this work, silver nanoparticles synthesized from *Cannabis sativa* extract exhibited MIC values ranging from 0.6 to 2.1 mg/L (Ali et al. 2018; Arokiyaraj et al. 2017; Feizi et al. 2018; Singh et al. 2014). Studies in the literature have consistently reported the inhibitory activity of silver nanoparticles against *Pseudomonas aeruginosa*, with MIC values ranging from 6.25 mg/l to 62.5 mg/l. Our results using Cannabis sativa extract for nanoparticle synthesis have shown superior antimicrobial efficacy, exhibiting notably lower MIC values ranging from 0.6 to 2.1 mg/l when compared to previously reported ranges.

Bactericidal effects of silver nanoparticles synthesized using “green” methods on adherent *P. aeruginosa* cells were also investigated in several experimental studies. In one study using industrially produced silver nanoparticles, the minimum bactericidal concentration (MBC) was determined as 4 mg/L. Another study using silver and silver chloride nanoparticles synthesized using *Malva sylvestris* leaf extract found MBC values ranging from 125 to 250 mg/L. Moreover, in a study utilizing silver nanoparticles synthesized from *Hibiscus sabdariffa* stem extract, the MBC was not found within the tested concentration range with maximum at 50 mg/L (Aboelmaati et al. 2021; Feizi et al. 2018; Markowska et al. 2014). While previous studies have reported varied bactericidal effects of silver nanoparticles against *P. aeruginosa* cells, with MBC values ranging from 4 mg/L for industrially produced nanoparticles to undetermined levels up to 50 mg/L for nanoparticles from *Hibiscus sabdariffa* stem extract, our results show a consistent bactericidal effect within the range of 2.1 to 9.6 mg/L. Notably, the MBC values obtained in our study, utilizing *Cannabis sativa* extracts, are comparable to those achieved with industrially produced nanoparticles.

Regarding gold nanoparticles produced using “green” methods, there is limited information in the literature about MIC or MBC values. In one study using gold nanoparticles synthesized using sodium tetraborohydrate, MIC was detected in the range of 16–24 g/L. Gold nanoparticles produced from *Ziziphus spina-christi* leaf extract exhibited inhibitory activity against *P. aeruginosa* with an MIC of 61 mg/L. Another study using gold nanoparticles synthesized with the use of fucoidan, a polysaccharide originating from brown algae, determined the MIC and MBC as 512 mg/L and 128 mg/L, respectively. The MBIC_50_ was reported in a study investigating the activity of gold nanoparticles synthesized using *Tinospora cordifolia* stem extract, with an MBIC_50_ value of 150 mg/L (Ali et al. 2020; Alzahrani et al. 2022; Khan et al. 2019; Zhao et al. 2010). In this work, gold nanoparticles exhibited lower efficacy compared to silver nanoparticles, with MIC_50_ values ranging from 59.6 to 159.7 mg/L and some strains (e.g., *P. aeruginosa* ATCC 15442) showing no MIC_50_ in the concentration range studied. MBIC_50_ was not found in most studied strains (e.g., *P. aeruginosa* DBM 3081 or ATCC 10145), and when found, it ranged from 7 to 98.4 mg/L. The variability in MIC and MBIC50 values for gold nanoparticles, as observed across various studies, underscores the significant impact of synthesis methods and materials on their antimicrobial effectiveness. Particularly, our findings of lower efficacy in certain *P. aeruginosa* strains highlight the complex interplay between the biological source of the synthesis material and the nanoparticles’ antimicrobial properties.

This study demonstrates the successful synthesis of silver and gold nanoparticles using agricultural waste extract from *Cannabis sativa*. These nanoparticles exhibited remarkable antimicrobial activity against *P. aeruginosa*, a common pathogen responsible for nosocomial infections and a major contributor to antibiotic resistance. The utilization of agricultural waste for nanoparticle production presents a sustainable and cost-effective approach. Developing nanoparticle coatings derived from such waste sources holds great potential for combating nosocomial infections, addressing antibiotic resistance, and offering affordable solutions for infection control in healthcare settings.

Future research should focus on optimizing the synthesis process to improve the antimicrobial properties and stability of these nanoparticles. Additionally, it is crucial to evaluate their efficacy in real-world applications, such as medical devices and wound dressings, to enable practical implementations. Leveraging the potential of nanoparticles derived from *Cannabis sativa* waste opens avenues for sustainable and accessible solutions in infection control and healthcare management.

## Conclusions

This study demonstrates the innovative use of *Cannabis sativa* waste extract for the sustainable synthesis of silver and gold nanoparticles, showcasing a cost-effective and eco-friendly approach to nanomaterial production. The successfully synthesized nanoparticles exhibit potent antimicrobial properties against *Pseudomonas aeruginosa* strains, which are known for their resistance to antibiotics and significance in nosocomial infections. This research highlights the potential of utilizing agricultural waste in developing effective antimicrobial agents, contributing to the fields of green nanotechnology and waste valorization within the circular economy. The findings offer promising directions for addressing antibiotic resistance and developing new strategies for infection control in healthcare settings.

### Limitations

While this study provides valuable insights into the green synthesis of silver and gold nanoparticles using *Cannabis sativa* waste extract and their antimicrobial properties, certain methodological limitations should be acknowledged. First, the chemical composition of *Cannabis sativa* waste extract may vary due to genetic differences between plant strains, environmental factors during cultivation, and variations in extraction methods. This variability could affect the reproducibility of nanoparticle synthesis and antimicrobial efficacy, necessitating further studies to standardize extraction protocols and characterize batch-to-batch differences. Second, the long-term stability of the synthesized nanoparticles was not evaluated. Future research should focus on assessing their physicochemical stability over time, including potential aggregation, oxidation, or loss of antimicrobial properties under different storage conditions.

Statistical analyses in this study confirmed significant antimicrobial effects, however future studies should include larger sample sizes and additional statistical models to further validate the observed trends. Additionally, the antimicrobial efficacy of the nanoparticles was assessed exclusively against *Pseudomonas aeruginosa* strains. While this bacterium is clinically relevant due to its antibiotic resistance and role in nosocomial infections, additional testing on Gram-positive bacteria, such as *Staphylococcus aureus*, and model organisms like *Escherichia coli* is needed to determine whether the observed effects extend to a broader range of bacterial pathogens. Moreover, this study focused on single-species biofilms. Future research should evaluate the efficacy of nanoparticles against multi-species biofilms, which better represent clinical conditions.

## Data Availability

The data underlying this article will be shared on reasonable request to the corresponding author.
